# Analysis of structure–activity and structure–mechanism relationships among thyroid stimulating hormone receptor binding chemicals by leveraging the ToxCast library†

**DOI:** 10.1039/d3ra04452a

**Published:** 2023-08-04

**Authors:** Ajaya Kumar Sahoo, Shanmuga Priya Baskaran, Nikhil Chivukula, Kishan Kumar, Areejit Samal

**Affiliations:** a The Institute of Mathematical Sciences (IMSc) Chennai 600113 India asamal@imsc.res.in; b Homi Bhabha National Institute (HBNI) Mumbai 400094 India

## Abstract

The thyroid stimulating hormone receptor (TSHR) is crucial in thyroid hormone production in humans, and dysregulation in TSHR activation can lead to adverse health effects such as hypothyroidism and Graves' disease. Further, animal studies have shown that binding of endocrine disrupting chemicals (EDCs) with TSHR can lead to developmental toxicity. Hence, several such chemicals have been screened for their adverse physiological effects in human cell lines *via* high-throughput assays in the ToxCast project. The invaluable data generated by the ToxCast project has enabled the development of toxicity predictors, but they can be limited in their predictive ability due to the heterogeneity in structure–activity relationships among chemicals. Here, we systematically investigated the heterogeneity in structure–activity as well as structure–mechanism relationships among the TSHR binding chemicals from ToxCast. By employing a structure–activity similarity (SAS) map, we identified 79 activity cliffs among 509 chemicals in TSHR agonist dataset and 69 activity cliffs among 650 chemicals in the TSHR antagonist dataset. Further, by using the matched molecular pair (MMP) approach, we find that the resultant activity cliffs (MMP-cliffs) are a subset of activity cliffs identified *via* the SAS map approach. Subsequently, by leveraging ToxCast mechanism of action (MOA) annotations for chemicals common to both TSHR agonist and TSHR antagonist datasets, we identified 3 chemical pairs as strong MOA-cliffs and 19 chemical pairs as weak MOA-cliffs. In conclusion, the insights from this systematic investigation of the TSHR binding chemicals are likely to inform ongoing efforts towards development of better predictive toxicity models for characterization of the chemical exposome.

## Abstract 2

Identification of activity cliffs as well as mechanism of action cliffs among TSHR binding chemicals from the ToxCast chemical library.

## Graphical abstract

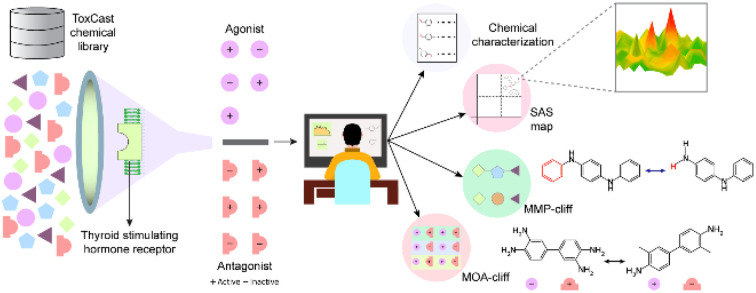

## Introduction

The thyroid stimulating hormone receptor (TSHR) plays an important role in the hypothalamic–pituitary–thyroid axis where it mediates the production of thyroid hormone upon activation by the physiologic agonist, thyroid stimulating hormone (TSH).^1–3^ The hypothalamic–pituitary–thyroid axis is crucial for development and metabolism, and is prone to disruption by endocrine disrupting chemicals (EDCs)^4–6^ in the human exposome. EDCs can bind to an endocrine receptor and dysregulate the hormonal activity in the human body, thus affecting the metabolism, immune system and reproductive system.^7^ In particular, animal studies have shown that EDCs binding to TSHR disrupt the thyroid system, ultimately leading to developmental toxicity.^8–10^ In humans, the overproduction of thyroid hormone caused by the binding of M22 autoantibody with TSHR can lead to Graves' disease,^11^ and underproduction of thyroid hormone caused by the binding of K1-70 autoantibody can lead to hypothyroidism and Hashimoto's disease.^12^ Consequently, screening of environmental chemicals in the human exposome that can bind to TSHR is important for their proper management.

The assessment of adverse effects of environmental chemicals on physiological targets is a laborious, time-consuming process and might involve animal testing. In this direction, the ToxCast program has screened nearly 10 000 chemicals for their adverse effects on various biological targets including TSHR, and characterized them based on their bioactivity and mechanisms of action.^13,14^ The ToxCast dataset has greatly enabled the development of several quantitative structure–activity relationship (QSAR) models that aim to predict toxicity of chemicals and aid in prioritization of chemicals for further testing.^15,16^ In particular, the ToxCast library has been used to develop machine learning based QSAR models to predict chemicals that bind to TSHR.^17,18^ However, the heterogeneity of the structure–activity landscape of chemicals that bind to TSHR has not been explored while developing such models, which could lead to uncertainties in associated predictions.^19^

The heterogeneity in the structure–activity landscape of chemicals arises due to the presence of activity cliffs.^20^ Activity cliffs are formed by chemical pairs that have similar structures but significantly differ in their activity values.^21^ The identification of activity cliffs in a chemical dataset is necessary as it limits the predictive power of QSAR models.^22^ Many methods have been developed for the analysis of the structure–activity landscape of chemicals and identification of activity cliffs.^23–27^ Medina-Franco and colleagues have extensively used the chemical fingerprint-based structure–activity similarity (SAS) map to identify activity cliffs in diverse chemical datasets.^28–30^ In an earlier contribution, some of us had extended this approach to identify and characterize activity cliffs in androgen receptor binding chemicals.^31^ Independently, Bajorath and colleagues have developed a substructure-based matched molecular pair (MMP) approach to identify activity cliffs.^32^ This approach has been extended by Hao *et al.*^33^ to identify the differences in the mechanisms of action of chemical pairs with similar structures, and moreover, introduced the concept of mechanism of action cliffs (MOA-cliffs). Like activity cliffs, the presence of MOA-cliffs highlights the heterogeneity in the structure–mechanism relationships among chemicals. Importantly, an exploration of the heterogeneity in the structure–activity landscape in conjunction with the structure–mechanism relationships has not been conducted on the ToxCast chemical library to date, in particular, for the chemicals that can bind to TSHR.

In this study, we performed a systematic investigation of the structure–activity landscape and structure–mechanism relationships in datasets of TSHR agonist and TSHR antagonist compiled from ToxCast chemical library. We employed both SAS map and MMP based approaches to identify the activity cliffs in the structure–activity landscape of these chemical datasets. We classified the identified activity cliffs into different categories using the information on their chemical structures. Further, we leveraged the mechanism of action (MOA) annotations for chemicals common to both TSHR agonist and TSHR antagonist datasets to identify MOA-cliffs. To the best of our knowledge, we present the first systematic study leveraging ToxCast chemical library and employing multiple cheminformatics approaches for the identification and characterization of activity cliffs along with MOA-cliffs among chemicals that can bind to TSHR.

## Methods

### Chemical dataset comprising of agonists and antagonists of the thyroid stimulating hormone receptor

**Fig. 1:**
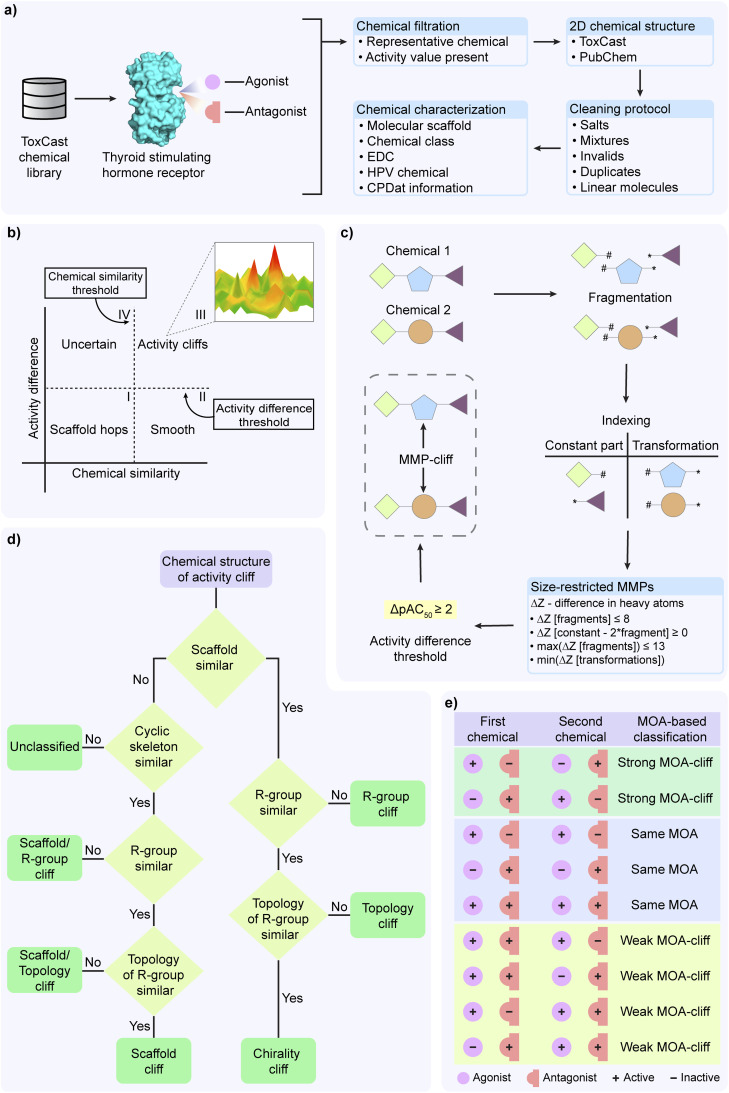
Summary of structure–activity landscape analysis and activity cliff identification in a chemical dataset curated from ToxCast library. (a) Curation and annotation of thyroid stimulating hormone receptor (TSHR) agonist and antagonist datasets. (b) Structure–activity similarity (SAS) map based approach to identify the activity cliffs in a chemical dataset. (c) Steps involved in generation of a matched molecular pair (MMP) and associated MMP-cliff. (d) Classification of activity cliff pairs based on respective structural information. (e) Mechanism of action (MOA) based classification of the chemical pairs (common to both TSHR agonist and antagonist datasets and having Tanimoto coefficient based similarity of >0.35) into three different categories.

The objective of this investigation is the analysis of the structure–activity landscape of the agonists and antagonists of the thyroid stimulating hormone receptor (TSHR) (Fig. 1). For this investigation, we retrieved the chemicals, their corresponding activity values, and endpoints from Tox21 assays (assay source identifier 7) within ToxCast version 3.5 (ref. 34) using level 5 and 6 processing. First, we used an in-house R script to filter the Tox21 multi-concentration summary file in order to identify chemicals based on their endpoint being either TSHR agonist (assay endpoint identifier 2040) or TSHR antagonist (assay endpoint identifier 2043) screened in HEK293T cell line. TSHR agonist is a chemical that binds to TSHR and fully activates it, whereas TSHR antagonist is a chemical that binds to TSHR but does not activate it and can additionally block the activation by any other agonist. Next, we filtered chemicals annotated as representative samples (*i.e.*, gsid_rep is 1) and with reported activity value (*i.e.*, modl_ga value is present) (Fig. 1a). Subsequently, for these shortlisted chemicals, we accessed the two-dimensional (2D) structures provided by ToxCast version 3.5, or PubChem (https://pubchem.ncbi.nlm.nih.gov/) if the 2D structures were not provided by ToxCast. Thereafter, we used MayaChemTools^35^ to remove salts, mixtures, invalid structures and duplicated chemicals (Fig. 1a). We also removed linear chemicals using the scaffold definition employed in our previous work.^31^ Finally, we curated a TSHR agonist dataset of 509 chemicals (ESI Table S1†) and a TSHR antagonist dataset of 650 chemicals (ESI Table S2†). For each chemical in the two datasets, we additionally compiled the Chemical Abstracts Service (CAS) registry number or PubChem compound identifiers, reported biological activity (*i.e.*, either active: hit_c is 1; or inactive: hit_c is 0), and the chemical concentration that generates the half maximal response (modl_ga, *i.e.*, logarithm of AC_50_ values in micromolar concentration).

### Molecular representation and annotation

We annotated chemicals in both TSHR agonist and TSHR antagonist datasets using molecular scaffolds and chemical classifications and their presence in different databases (Fig. 1a). Following our previous work,^31^ we used the Bemis–Murcko definition^36^ to compute the molecular scaffolds from chemical structures. Next, we relied on ClassyFire^37^ to provide the corresponding chemical classifications. Further, we used DEDuCT^38,39^ database which compiles information on 792 endocrine disrupting chemicals (EDCs) curated from published literature with supporting evidence for endocrine disruption from experiments in humans and rodents, to identify the known EDCs among chemicals in the TSHR agonist or TSHR antagonist dataset. We also used Organisation for Economic Co-operation and Development High Production Volume (OECD HPV) (https://www.oecd.org/chemicalsafety/risk-assessment/33883530.pdf) or United States High Production Volume (USHPV) (https://comptox.epa.gov/dashboard/chemical-lists/EPAHPV) databases to identify high production volume chemicals in our datasets. Additionally, we leveraged the CAS identifiers of the chemicals in TSHR agonist and TSHR antagonist datasets, which are also compiled in Distributed Structure-Searchable Toxicity (DSSTox) database, to retrieve annotations such as functional uses, occupational health hazard reports and product use composition from Chemical and Products Database (CPDat) (Fig. 1a).^40^

### Computation of activity difference

The activity difference for a pair of chemical is considered as the difference between their corresponding pAC_50_ values, where pAC_50_ is the negative logarithm of AC_50_ value in molar concentration.^28,33,41^ The activity values of the chemicals in the compiled TSHR agonist and TSHR antagonist datasets are reported as the logarithm of AC_50_ values in micromolar concentrations (modl_ga). Therefore, we converted the modl_ga value to pAC_50_ value using the following formulae:AC_50_(M) = 10^modl_ga^ × 10^−6^pAC_50_ = −log_10_(AC_50_(M)) = 6 − modl_ga

Thereafter, we calculated the activity difference between two chemicals *i* and *j* using the following formula:Activity difference = |(pAC_50_)_*i*_ − (pAC_50_)_*j*_|wherein the (pAC_50_)_*i*_ and (pAC_50_)_*j*_ are the pAC_50_ values of chemicals *i* and *j* respectively.

### Identification of activity cliffs using structure–activity similarity (SAS) map

We independently analyzed the activity landscape of the chemicals in TSHR agonist and TSHR antagonist datasets using structure–activity similarity (SAS) map (Fig. 1b).^28–31^ SAS map is a 2D representation where the structural similarity between the chemicals is plotted along the *x*-axis and the activity difference between the chemicals is plotted along the *y*-axis (Fig. 1b). We computed structural similarity between chemical pairs based on Tanimoto coefficient between the corresponding extended-connectivity fingerprints with diameter 4 (ECFP4) of the chemicals. As there is no strict rule to choose a threshold for high structural similarity,^42^ we considered a similarity threshold of 0.35 which was close to three standard deviations from median of the computed Tanimoto coefficient for chemical pairs in both TSHR agonist and TSHR antagonist datasets. We considered an activity difference threshold of 100 fold change which is equivalent to 2 logarithmic units. The scaffold hops region (region I in Fig. 1b) corresponds to the chemicals which are structurally different but activity-wise similar. The smooth region (region II in Fig. 1b) corresponds to chemicals which are structurally similar and activity-wise also similar. The uncertain region (region IV in Fig. 1b) corresponds to chemicals which are structurally different and activity-wise also different. Importantly, we designated the highly similar chemical pairs (Tanimoto coefficient > 0.35) with high activity difference (≥2) as the activity cliffs in both TSHR agonist and TSHR antagonist datasets (region III in Fig. 1b). Additionally, we considered chemicals which form at least 5 activity cliff pairs as activity cliff generators (ACGs).^29,31^

### Identification of activity cliffs based on matched molecular pairs (MMPs)

In addition to SAS map based activity landscape analysis, we employed the matched molecular pairs (MMP) based approach to identify the activity cliffs (MMP-cliffs)^32^ independently in TSHR agonist and TSHR antagonist datasets (Fig. 1c). We used mmpdb platform^43^ to generate MMPs for chemicals in both datasets. First, the mmpdb fragment module was used to fragment the chemical structure with ‘none’ value for both maximum number of non-hydrogen atoms and maximum number of rotatable bonds arguments. Next, the mmpdb index module was used to generate an exhaustive list of MMPs with ‘none’ value for maximum number of non-hydrogen atoms in the variable fragment argument. This gave us an exhaustive list of MMPs with detailed information on the constant part and transformations containing the exchanged fragments between chemical pairs. Further, to generate size-restricted MMPs, we implemented the following four criteria (Fig. 1c):^32^

(i) The difference in number of heavy atoms of the exchanged fragments in transformation should not exceed 8.

(ii) The constant part should be at least twice the size of each fragment in the transformation.

(iii) The number of heavy atoms of each fragment in the transformation should not exceed 13.

(iv) For a chemical pair with multiple MMPs, the transformation with the least difference in the number of heavy atoms between the exchanged fragments is considered.

Finally, we identified MMP-cliffs among the size-restricted MMPs by selecting those pairs with an activity difference ≥ 2 in logarithmic units (*i.e.*, 100 fold change) (Fig. 1c).

### Activity cliff classification

In this study, we followed the activity cliff classification described in Vivek-Ananth *et al.*,^31^ to classify the activity cliffs by considering their molecular scaffolds, R-groups, R-group topology, and chirality of chemical structures. Further, we modified the workflow in Vivek-Ananth *et al.*^31^ to also check for topologically equivalent scaffolds (cyclic skeleton) when a pair of chemicals do not share the same scaffolds (Fig. 1d).^24^ We used the R-group decomposition module available in RDKit^44^ to decompose the chemical structure into its core structure (scaffold) and R-groups. Further, we used the modified workflow (Fig. 1d) to manually classify the activity cliffs into the following 7 types:

(i) Chirality cliff: chemical pairs having the same scaffold, R-groups and R-group topology.

(ii) Topology cliff: chemical pairs having different R-group topologies while their scaffolds and R-groups remain unchanged.

(iii) R-group cliff: chemical pairs having different R-groups while their scaffolds remain unchanged.

(iv) Scaffold cliff: chemical pairs having different scaffolds while their cyclic skeletons, R-groups and R-group topologies remain unchanged.

(v) Scaffold/topology cliff: chemical pairs having different scaffolds and R-group topologies while their cyclic skeletons and R-groups remain unchanged.

(vi) Scaffold/R-group cliff: chemical pairs having different scaffolds and R-groups while their cyclic skeletons remain unchanged.

(vii) Unclassified: chemical pairs having different scaffolds and cyclic skeletons.

### Mechanism of action (MOA) based classification of chemical structures

In addition to the activity cliffs in TSHR agonist and TSHR antagonist datasets, we were interested in identifying chemical pairs in which the chemicals have similar structures but differ in their mechanism of action (MOA). Such chemical pairs are designated as MOA-cliffs.^33^ We considered chemicals which were common to both the TSHR agonist and TSHR antagonist datasets, and removed those chemicals which were reported as inactive MOA in both assays. We then computed the structural similarity of chemical pairs by using the Tanimoto coefficients between the ECFP4 fingerprints of the shortlisted chemicals. We chose 0.35 as the similarity threshold (which is the structural similarity threshold used in SAS map analysis) to filter similar chemical pairs. Based on their MOA annotations in TSHR agonist and TSHR antagonist datasets, we classified these chemical pairs into 3 types (Fig. 1e):

(i) Strong MOA-cliff: chemical pairs in which the chemicals have opposite MOA annotations.

(ii) Same MOA: chemical pairs in which both the chemicals have same MOA annotations.

(iii) Weak MOA-cliff: chemical pairs which could not be classified as either strong MOA-cliff or same MOA.

## Results and discussion

### Exploration of the chemical space of TSHR agonist and antagonist datasets

From ToxCast library, we curated 509 chemicals in TSHR agonist (ESI Table S1†) and 650 chemicals in TSHR antagonist (ESI Table S2†) datasets, and thereafter, annotated the chemicals in the two datasets with information on their molecular scaffolds, chemical classifications, and their presence in public documentation or databases (Methods; Fig. 1a). Notably, there were 89 chemicals common between TSHR agonist and TSHR antagonist datasets. Additionally, we observed that chemicals in both TSHR agonist and TSHR antagonist datasets are structurally diverse (median Tanimoto coefficient based similarity using ECFP4 fingerprints of ∼0.11), which could be attributed to the diverse composition of environmental chemicals in the ToxCast chemical library, which are assessed for their adverse biological effects.^13,15^

For the 509 chemicals in the TSHR agonist dataset, after computing the molecular scaffolds we observed that the benzene scaffold is highly represented (as it is found in 122 chemicals). Many of the chemicals in TSHR agonist dataset are also categorized under the chemical class of ‘benzene and substituted derivatives’ (195 chemicals) (ESI Table S1†). Importantly, 79 chemicals in the TSHR agonist dataset are documented in DEDuCT^38,39^ as endocrine disrupting chemicals (EDCs) with experimental evidence, of which 29 EDCs have category II evidence (supporting evidence from *in vivo* rodent and *in vitro* human experiments but not from *in vivo* human experiments), 28 EDCs have category III evidence (supporting evidence from only *in vivo* rodent experiments), 21 EDCs have category IV evidence (supporting evidence from only *in vitro* human experiments) and 1 EDC has category I evidence (supporting evidence from *in vivo* human experiments). Among the 79 identified EDCs, 21 chemicals are also documented as high production volume chemicals as per OECD HPV or USHPV databases (Methods; ESI Table S1†). Chemical and Products Database (CPDat) provided various functional use annotations for 102 chemicals, of which biocides, fragrance and antioxidants are the major reported functional categories (ESI Table S1†). CPDat also provided the product use composition data for 70 chemicals, of which personal care, and cleaning products and household care are the major categories (ESI Table S1†). Additionally, 4 chemicals namely, 3-carene, butylated hydroxytoluene, hydroquinone and triphenyl phosphate have been documented in various occupational health hazard reports (ESI Table S1†).

Similarly, for the 650 chemicals in the TSHR antagonist dataset, we observed that benzene scaffold is the most represented molecular scaffold (as it is found in 127 chemicals), while ‘benzene and substituted derivatives’ is the most represented chemical class (254 chemicals) (ESI Table S2†). Notably, 65 chemicals in the TSHR antagonist dataset are documented as EDCs in DEDuCT, of which 26 EDCs have category III evidence (supporting evidence from only *in vivo* rodent experiments), 22 EDCs have category II evidence (supporting evidence from *in vivo* rodent and *in vitro* human experiments but not from *in vivo* human experiments) and 17 EDCs have category IV evidence (supporting evidence from only *in vitro* human experiments). Among the 65 identified EDCs, 13 are also documented as high production volume chemicals in OECD HPV or USHPV databases (ESI Table S2†). CPDat provided functional uses for 156 chemicals, of which biocides, fragrance and antioxidants are reported as the major functional categories (ESI Table S2†). CPDat also provided the product use composition data for 107 chemicals, of which personal care, pesticides, and cleaning products and household care are the major categories (ESI Table S2†). Additionally, 4 antagonists namely, 2,2′,4,4′,5-pentabromodiphenyl ether, 2,2′,4,4′-tetrabromodiphenyl ether, bibenzyl and styrene are documented in various occupational health hazard reports (ESI Table S2†).

### Activity landscape analysis of TSHR agonist dataset

**Fig. 2:**
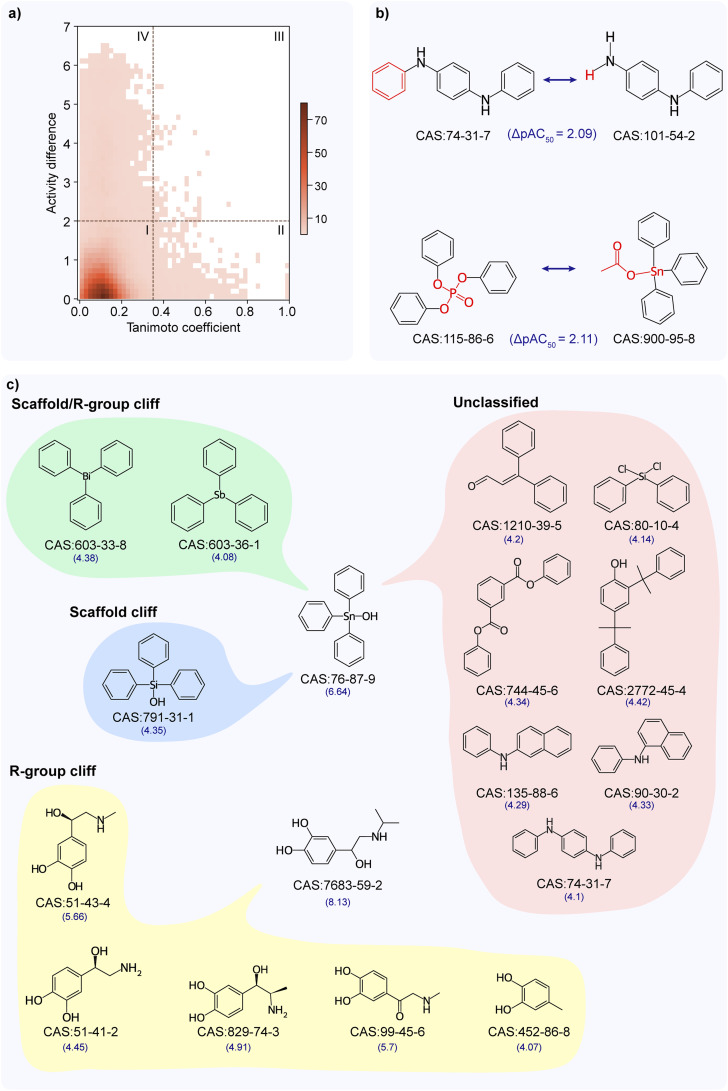
Activity landscape analysis of TSHR agonist dataset. (a) Structure–activity similarity (SAS) map for TSHR agonist dataset. SAS map is divided into 4 quadrants by considering a similarity threshold of 0.35 and activity difference threshold of 2. Further, the density of data points in different regions of the SAS map is shown using a color gradient. (b) MMP-cliffs formed by *N*,*N*′-diphenyl-*p*-phenylenediamine (CAS identifier 74-31-7) with *N*-phenyl-1,4-benzenediamine (CAS identifier 101-54-2) [ΔpAC_50_ = 2.09] and triphenyl phosphate (CAS identifier 115-86-6) with triphenyltin acetate (CAS identifier 900-95-8) [ΔpAC_50_ = 2.11]. The transformed fragments resulting in MMP-cliff are highlighted in red color. (c) Activity cliff classifications for the activity cliff generators, triphenyltin hydroxide (CAS identifier 76-87-9; 10 activity cliff pairs) and isoproterenol (CAS identifier 7683-59-2; 5 activity cliff pairs). The activity value (pAC_50_) is mentioned below for each chemical.

The structure–activity similarity (SAS) map has been employed in the literature to identify activity cliffs by investigating the structure–activity relationship.^28–31^ Accordingly, we analyzed the activity landscape of the TSHR agonist dataset using the SAS map approach (Methods; Fig. 2a). We observed that the majority of chemical pairs show similar activity while they are structurally diverse (see SAS map region 1 in Fig. 2a). Importantly, we identified 79 chemical pairs showing high activity difference while being structurally similar (see SAS map region III in Fig. 2a). We designated these 79 chemical pairs (formed by 60 unique chemicals) as activity cliffs (ESI Table S3†), of which 9 chemicals are additionally identified as activity cliff generators (ACGs) (Methods; ESI Table S4†). The chemicals forming activity cliffs are represented by 34 unique scaffolds with benzene and triphenyltin scaffolds being the highly represented scaffolds, and are categorized under 13 chemical classes with ‘benzene and substituted derivatives’ class being the largest category. Moreover, triphenyltin scaffold is highly represented in chemicals forming ACGs. The chemicals forming pairs in the region I (scaffold hops) and region IV (unknown) are dominated by ‘benzene and substituted derivatives’ chemical class followed by ‘prenol lipids’ chemical class. Similarly, the chemicals forming pairs in the region II (smooth) are dominated by ‘benzene and substituted derivatives’ chemical class followed by ‘steroids and steroid derivatives’ chemical class.

Matched Molecular Pair (MMP) based activity landscape analysis has been alternatively employed in the literature to identify the activity cliffs.^32,33^ We also used the MMP approach to analyze the activity landscape of the TSHR agonist dataset. We identified 523 MMPs formed by 170 chemicals in the TSHR agonist dataset (Methods; ESI Table S5†), of which 38 MMPs (formed by 19 unique chemical pairs) are identified as MMP-cliffs based on an activity difference threshold consideration similar to SAS map (Methods; ESI Table S3†). Notably, the MMP-cliffs identified by the MMP approach are a subset of the activity cliffs identified by the SAS map approach, which could be attributed to the highly restrictive fragment transformation conditions imposed in the generation of MMPs.^32^ Interestingly, the constant part containing three benzene rings identified in 14 of the 38 MMP-cliffs is similar to the highly represented triphenyltin scaffold among the chemicals forming activity cliffs identified through SAS map. Fig. 2b shows chemical pairs of *N*,*N*′-diphenyl-*p*-phenylenediamine (CAS identifier 74-31-7) and *N*-phenyl-1,4-benzenediamine (CAS identifier 101-54-2), triphenyl phosphate (CAS identifier 115-86-6) and triphenyltin acetate (CAS identifier 900-95-8) that are identified as MMP-cliffs. *N*,*N*′-Diphenyl-*p*-phenylenediamine is an ACG which is documented as an EDC in DEDuCT and present in the OECD HPV or USHPV databases. Notably, triphenyl phosphate and triphenyltin acetate are documented as EDCs in DEDuCT and triphenyl phosphate is also present in the OECD HPV or USHPV databases.

Subsequently, we classified the 79 activity cliffs and identified 11 as R-group cliffs, 1 as scaffold cliff, 11 as scaffold/R-group cliffs and 56 as unclassified (Methods; ESI Table S3†). Fig. 2c shows the different classifications of the activity cliffs formed by triphenyltin hydroxide (CAS identifier 76-87-9) and isoproterenol (CAS identifier 7683-59-2). Triphenyltin hydroxide forms 10 activity cliff pairs where 2 are scaffold/R-group cliffs (same cyclic skeleton but differ in the scaffold as well as R-group), 1 is scaffold cliff (same R-group, R-group topology and cyclic skeleton but differ only in scaffold) and remaining are unclassified (differ in scaffold as well as the cyclic skeleton). Similarly, isoproterenol forms 5 activity cliff pairs where all are R-group cliffs (same scaffold and cyclic skeleton but differ in R-groups). Further, we noted that majority of the identified activity cliffs (56 of 79) are classified under the unclassified category as the chemicals forming these cliffs differ in their scaffolds as well as their scaffold topology (cyclic skeleton).

### Activity landscape analysis of TSHR antagonist dataset

**Fig. 3:**
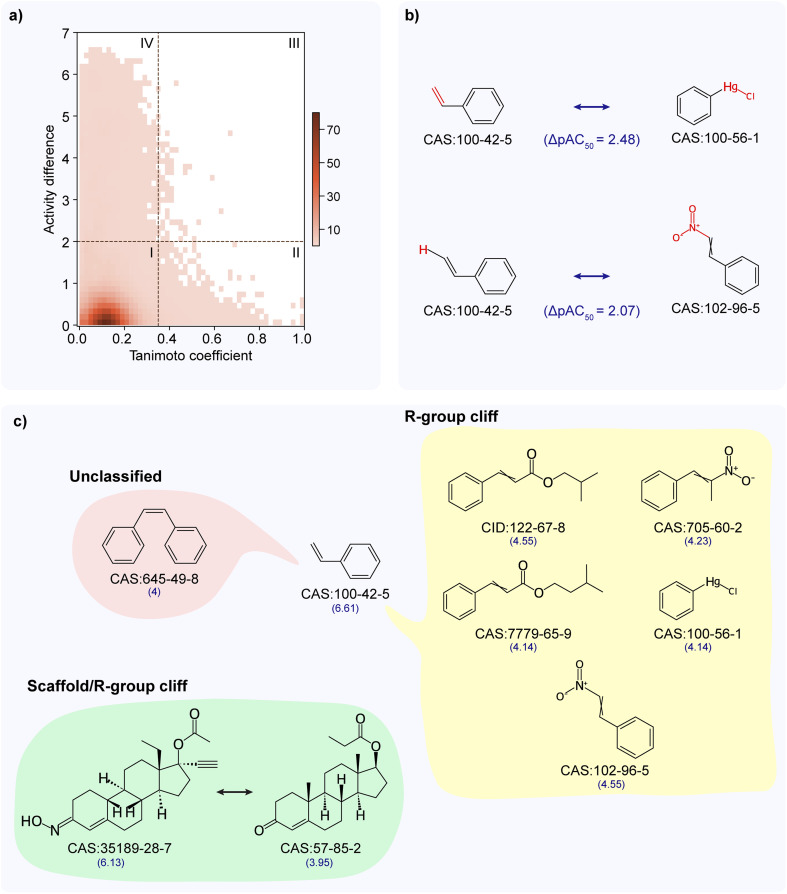
Activity landscape analysis of TSHR antagonist dataset. (a) Structure–activity similarity (SAS) map for TSHR antagonist dataset. SAS map is divided into 4 quadrants by considering a similarity threshold of 0.35 and activity difference threshold of 2. Further, the density of data points in different regions of the SAS map is shown using a color gradient. (b) MMP-cliffs formed by styrene (CAS identifier 100-42-5) with phenylmercuric chloride (CAS identifier 100-56-1) [ΔpAC_50_ = 2.48] and with beta-nitrostyrene (CAS identifier 102-96-5) [ΔpAC_50_ = 2.07]. The transformed fragments resulting in MMP-cliff are highlighted in red color. (c) Activity cliff classifications for the activity cliff generator, styrene (6 activity cliff pairs) and an activity cliff pair of norgestimate (CAS identifier 35189-28-7) with testosterone propionate (CAS identifier 57-85-2). The activity value (pAC_50_) is mentioned below for each chemical.

Similar to the activity landscape analysis of the TSHR agonist dataset, we analyzed the TSHR antagonist dataset through both SAS map and MMP approaches. From the SAS map approach, while most chemical pairs show similar activity despite having diverse structures (see SAS map region I in Fig. 3a), 69 chemical pairs showed high activity difference while they are structurally similar (see SAS map region III in Fig. 3a). We designated these 69 chemical pairs as activity cliffs, and observed that they are formed by 75 unique chemicals (ESI Table S6†), of which 4 chemicals are ACGs (Methods; ESI Table S7†). The chemicals forming activity cliffs are represented by 39 unique scaffolds with benzene and biphenyl scaffolds being the highly represented scaffolds, and are categorized under 17 chemical classes with ‘benzene and substituted derivatives’ class being the largest category. Similar to the activity cliff region, chemicals forming pairs in other three regions (region I, II and IV) are also dominated by ‘benzene and substituted derivatives’ chemical class followed by ‘steroids and steroid derivatives’ chemical class.

From the MMP approach, we identified 590 MMPs (formed by 195 chemicals), of which 3 are MMP-cliffs (Methods; ESI Table S8†). Notably all the MMP-cliffs are also activity cliffs identified through SAS map approach. Fig. 3b shows chemical pairs of styrene (CAS identifier 100-42-5) and phenylmercuric chloride (CAS identifier 100-56-1), and styrene and beta-nitrostyrene (CAS identifier 102-96-5). Styrene is an ACG which is documented as an EDC in DEDuCT and present in the OECD HPV or USHPV databases.

Further, we classified the 69 activity cliffs and identified 18 as R-group cliffs (same R-group but differ in scaffold), 1 as scaffold/R-group cliff (same cyclic skeleton but differ in both scaffold and R-group) and 50 as unclassified (differ in both scaffold and cyclic skeleton) (Methods; ESI Table S6†). Fig. 3c shows 6 activity cliffs formed by styrene, 5 R-group cliffs, and 1 unclassified (differ in both scaffold and cyclic skeleton) and 1 scaffold/R-group cliff formed by norgestimate (CAS identifier 35189-28-7) and testosterone propionate (CAS identifier 57-85-2). Finally, similar to the activity cliff classification in the TSHR agonist dataset, we noted that majority of the activity cliffs in the TSHR antagonist dataset (50 of 69) are classified under the unclassified category.

### Mechanism of action (MOA) cliffs

**Fig. 4:**
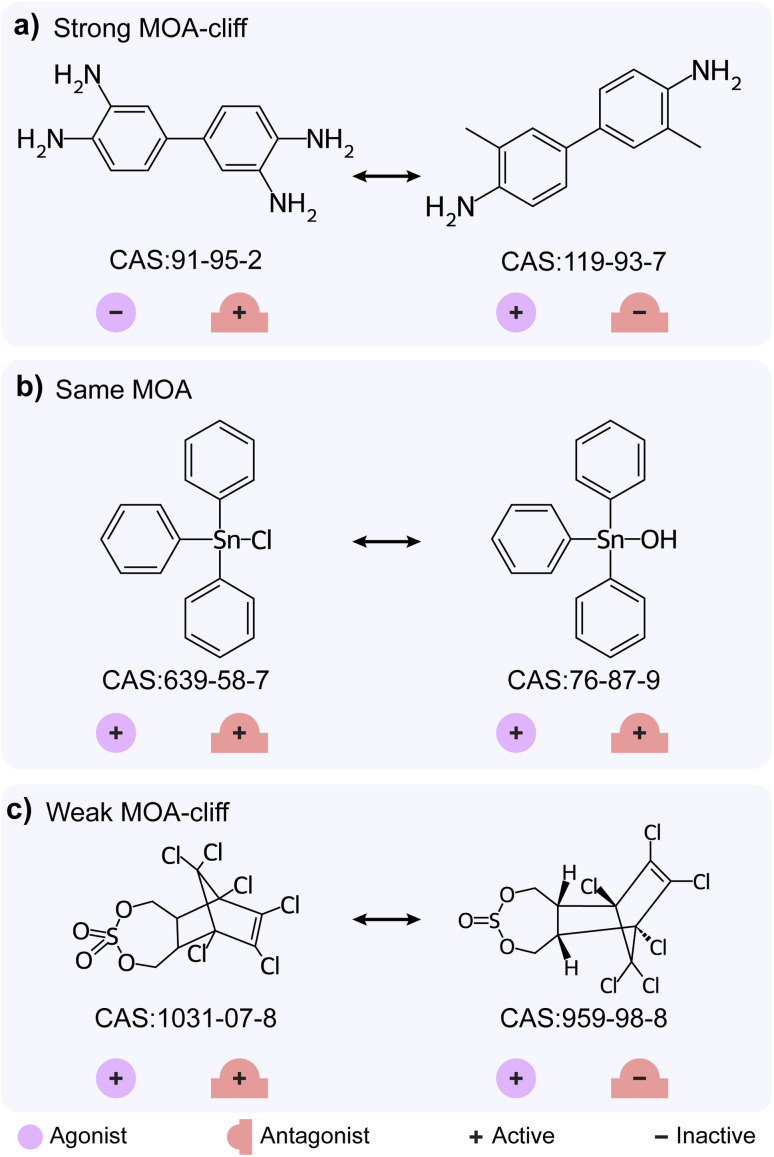
Examples for three different mechanism of action (MOA) based classifications of chemical pairs. (a) Strong MOA-cliff formed by 3,3′-diaminobenzidine (CAS identifier 91-95-2) with 3,3′-dimethylbenzidine (CAS identifier 119-93-7). (b) Same MOA formed by triphenyltin chloride (CAS identifier 639-58-7) with triphenyltin hydroxide (CAS identifier 76-87-9). (c) Weak MOA-cliff formed by endosulfan sulfate (CAS identifier 1031-07-8) with endosulfan I (CAS identifier 959-98-8).

Apart from the differences in activity, structurally similar chemicals also show a difference in their identified mechanism of action (MOA). Hao *et al.*^33^ have earlier explored the MMPs with different MOAs from androgen receptor agonist and antagonist datasets, and designated them as MOA-cliffs. We shortlisted 75 chemicals which have endpoints in both TSHR agonist and TSHR antagonist datasets and identified 38 chemical pairs which have high structural similarity (Methods; ESI Table S9†). We classified these 38 chemical pairs based on their MOA annotations and identified 3 as strong MOA-cliffs, 16 as same MOA and 19 as weak MOA-cliffs (Methods; Fig. 1e; ESI Table S9†). Notably, 1 strong MOA-cliff and 8 weak MOA-cliffs are also classified as activity cliffs identified through the SAS map approach. Fig. 4 shows examples of different MOA based classifications of highly similar chemical pairs (Tanimoto coefficient > 0.35). 3,3′-Diaminobenzidine (CAS identifier 91-95-2; inactive agonist and active antagonist) and 3,3′-dimethylbenzidine (CAS identifier 119-93-7; active agonist and inactive antagonist) form strong MOA-cliff, triphenyltin chloride (CAS identifier 639-58-7; active agonist and active antagonist) and triphenyltin hydroxide (CAS identifier 76-87-9; active agonist and active antagonist) form same MOA, and endosulfan sulfate (CAS identifier 1031-07-8; active agonist and active antagonist) and endosulfan I (CAS identifier 959-98-8; active agonist and inactive antagonist) form weak MOA-cliff.

## Conclusions

In this study, we explored and analyzed the activity landscape of chemicals in curated datasets of thyroid stimulating hormone receptor (TSHR) agonists (TSHR agonist dataset) and antagonists (TSHR antagonist dataset) compiled from the ToxCast library. By leveraging the established fingerprint-based approach and a substructure-based approach, we identified 79 activity cliffs in the TSHR agonist dataset and 69 activity cliffs in the TSHR antagonist dataset. Furthermore, we classified the resultant activity cliffs based on the information on chemical structures. Additionally, we analyzed the differences in the mechanism of action (MOA) of the TSHR binding chemicals and identified 3 strong MOA-cliffs and 19 weak MOA-cliffs based on the difference in their annotated bioactivity outcomes. Notably, this is the first study to report the heterogeneity of the structure–activity landscape as well as the structure–mechanism relationships of the TSHR binding chemicals compiled from ToxCast chemical library.

However, our workflow does not account for the stereoisomeric information of the chemical structures in identification of activity cliffs and MOA-cliffs. Moreover, we were unable to quantify the differences in binding affinities of chemicals forming MOA-cliffs as their affinity values are obtained from two different assays. We were also unable to explore molecular mechanisms behind the formation of activity cliffs as well as MOA-cliffs as there are no experimentally determined co-crystallized TSHR protein–ligand complexes available in the public domain.

Nonetheless, our efforts highlight the presence of activity cliffs and MOA-cliffs in a large chemical dataset such as ToxCast, and their identification will aid in development of robust toxicity predictors.^22,45^ In the future, one can use the newly developed chemical similarity methods such as extended similarity indices (*n*-ary comparison)^46,47^ to deal with the computational complexity arising from pairwise comparison for large chemical datasets. In conclusion, this is the first investigation that combines SAS map and MMP approaches along with large-scale datasets from ToxCast chemical library to identify and characterize activity cliffs and MOA-cliffs among TSHR agonist and TSHR antagonist datasets. We believe that these insights will aid in development of better toxicity prediction models, and thereby, contribute towards characterization of the human exposome.

## Author contributions

Ajaya Kumar Sahoo: conceptualization, data compilation, data curation, formal analysis, software, visualization, writing; Shanmuga Priya Baskaran: conceptualization, data compilation, data curation, formal analysis, software, visualization, writing; Nikhil Chivukula: data compilation, data curation, formal analysis, writing; Kishan Kumar: formal analysis, visualization; Areejit Samal: conceptualization, supervision, formal analysis, writing.

## Conflicts of interest

The authors declare that they have no known competing financial interests or personal relationships that could have appeared to influence the work reported in this paper.

## Supplementary Material

RA-013-D3RA04452A-s001
